# Species Identification in the *Rhododendron vernicosum*–*R. decorum* Species Complex (Ericaceae)

**DOI:** 10.3389/fpls.2021.608964

**Published:** 2021-01-28

**Authors:** Xingxing Mao, Ji Wang, Nawal Shrestha, Yazhen Ma, Jianquan Liu

**Affiliations:** ^1^Key Laboratory of Bio-Resource and Eco-Environment of Ministry of Education and State Key Lab of Hydraulics and Mountain River Engineering, College of Life Sciences, Sichuan University, Chengdu, China; ^2^State Key Laboratory of Grassland Agro-Ecosystem, Institute of Innovation Ecology, Lanzhou University, Lanzhou, China

**Keywords:** species delimitation, *Rhododendron*, SSR, morphological analyses, hybridization

## Abstract

Delimitating species boundaries is the primary aim of biological classification and could be critical for evaluating the evolving process of species and conserving biodiversity. *Rhododendron* is an iconic group with an extraordinary diversity in southwest China. However, it remains unknown whether the recorded species therein comprise independently evolving lineages or artificially delimitated morphological entities. In this study, we carried out species delimitation of four *Rhododendron* species in the *R. vernicosum*-*R. decorum* species complex based on morphological analyses and population genetic data from nuclear simple sequence repeats (SSR) markers. We randomly selected a total of 105 specimens of different individuals identified as four species across their distributional ranges to examine the statistically distinct phenotypic clusters based on 19 morphological traits. Similarly, we genotyped 55 individuals of four species from 21 populations using 15 SSR markers. The morphological analyses sorted *R. decorum* and the other three species into two different phenotypic clusters. The genetic clusters were consistent with the morphological clusters. However, we also recovered the third genetic cluster, comprising six *R. vernicosum* populations and containing the admixed genetic compositions of the other two distinct genetic clusters. This hybrid group was morphologically similar to the typical *R. vernicosum* (including the samples from its type specimen locality and both *R. verruciferum* and *R. gonggashanense*) but with more genetic ancestry from *R. decorum*. Based on our findings, we identify two distinct species and one putative hybrid group due to introgression in the *R. vernicosum-R. decorum* species complex. We propose to merge *R. verruciferum* and *R. gonggashanense* into *R. vernicosum* based on genetic compositions and our morphological analyses. The hybrid group inferred from our findings, however, needs further investigations.

## Introduction

Accurate species delimitation is crucial for biodiversity conservation because an error in species recognition will result in wasted effort in species conservation and may cause erroneous scientific inference when using species as the basic analysis unit (Mallet, 1995; De Queiroz, 2007; Wiens, 2007). This is particularly difficult for some taxonomically complicated plant groups that comprise numerous closely related species (Rieseberg et al., 2006). Two widespread factors may likely lead to wrong species delimitations (Petit and Excoffier, 2009; Hu et al., 2015; Liu et al., 2015; Wang et al., 2018; Li et al., 2020). First, intraspecific morphological variations may lead to the recognition of conspecific taxa as different species, but these variations within species usually form a single morphological cluster under systematic statistical analyses (Su et al., 2015, 2017; Klishko et al., 2018). Second, interspecific hybridization and introgression result in the production of hybrid populations or individuals (Petit and Excoffier, 2009), and some hybrids may develop into a new hybrid species or be on the way to becoming independently evolving species (Liu, 2016). However, individuals with cryptic introgressions and without obvious morphological transitions are still difficult to be recovered and taxonomically treated. Despite that multiple approaches under different species concepts were proposed (Sokal and Sneath, 1963; Cracraft, 1983; Wiens and Penkrot, 2002; Ramsey et al., 2003; Rissler and Apodaca, 2007; Craven et al., 2008; Yang and Rannala, 2010; Niemiller et al., 2011; Reeves and Richards, 2011; Hu et al., 2015; Liu et al., 2015), it is better to carry out species delimitations based on the consensus that different species should represent different evolving lineages, and morphological statistics and population genotyping seem to be the two convenient but necessary approaches for such a study (Liu, 2016).

In this study, we aimed to delimitate closely related species in a large and taxonomically difficult plant group, *Rhododendron* L. Most of the recorded 1,000 species in this genus are distributed in southwest China (Feng, 1983; Min, 1984; Min and Fang, 1990; Fang, 1992, 1993; Chamberlain et al., 1996; Goetsch et al., 2005; Fang et al., 2005; Geng, 2016). However, a few published species may be established based on the interspecific hybrids rather than independently evolving lineages and statistical morphological clustering because hybridization and cryptic introgression with obvious morphological changes prevail in this genus, especially in the southwestern Chinese species (Milne et al., 1999, 2003, 2010; Milne and Abbott, 2008; Ma et al., 2010; Zha et al., 2010; Marczewski et al., 2015; Yan et al., 2015; Wang et al., 2018). Because of the recent diversification of *Rhododendron*, previous studies based on DNA barcodes (chloroplast DNAs and ITS) failed to diagnose the closely related species and detect hybridization and introgression due to the lack of distinct genetic divergences in these DNA sequences (Leavitt et al., 2013; Zeng et al., 2013; Divakar et al., 2015; Li et al., 2015; Yan et al., 2015). However, nuclear simple sequence repeats (SSR) genotyping approach with more abundant genetic variations between individuals, populations and species, seems to be a better alternative for such an aim (Wang et al., 2018). Therefore, we used this method and morphological statistics to delimitate the species boundary for a species complex in the subsection *Fortunea* of the subgenus *Hymenanthes*. This complex was initially named here as “the *R. vernicosum*-*R. decorum* species complex” for narrative convenience and taxonomical relatedness, containing the four species, *Rhododendron vernicosum* Franch., *R. verruciferum* W. K. Hu, *R. gonggashanense* W. K. Hu and *R. decorum* Franch. In previous studies, *R. verruciferum* and *R. gonggashanense* were considered to be more closely related to *R. decorum* (Hu, 1988a,b). However, both species are sympatric to *R. vernicosum* without distinct geographic isolations and morphologically similar to *R. vernicosum*, besides, they are alike to each other. We explored all the recorded populations and collected representative individuals to address the following two questions: (1) Do *R. verruciferum* and *R. gonggashanense* comprise two independent morphological and genetic clusters? and (2) Did hybridization and cryptic introgression occur under the similar morphology in this species complex?

## Materials and Methods

### Taxonomy, Morphological Chazracteristics, and Distribution of Study Species

The four species in the *Rhododendron vernicosum*-*R. decorum* species complex belong to subsection *Fortunea* within the section *Ponticum* of the subgenus *Hymenanthes* based on the traditional taxonomy (Chamberlain et al., 1996; Fang et al., 2005; Goetsch et al., 2005; Geng, 2016). These four species together could be distinguished from other related species by broadly funnel-campanulate corollas with 6–7 lobes, 12–16 stamens with white indumentum in the basal segment, rough pedicle, ovary and style with red or white glands, and inconspicuous calyx with glands (Hu and Fang, 1994; Fang et al., 2005). The discriminations between four species remain in debate although inflorescence rachis, leaf morphology and stigma size were used to distinguish them in the previous taxonomic descriptions. For example, *R. decorum* was described to have bigger stigmas than other threes (Fang et al., 2005), but one of its subspecies (*R. decorum* subsp. *parvistigmaticum*) was described to also have small stigmas. In addition, differences from leaf base, gland color, glandular hairs of pedicles, and styles and corolla size were used to distinguish *R. vernicosum*, *R. verruciferum*, and *R. gonggashanense* (Hu, 1988a,b; Hu and Fang, 1994; Fang et al., 2005). While *R. vernicosum* was described to have pink and white corolla, leaf blades with a round or nearly round base and pedicles or styles with red glands, and *R. verruciferum* has smaller leaf blades and corolla size, but also has pedicles or styles with glands or glandular hairs and white-pink corolla that is particularly similar to *R. vernicosum*. Meanwhile, *R. gonggashanense* was described to have leaf blades with round or slightly cordate base and white corolla that is mostly overlapped with *R. vernicosum* as well. Besides, the distributional ranges of these four species are largely overlapped based on the previous reports (Min, 1984; Fang and Hu, 1986; Hu, 1988a,b; Hu and Fang, 1994; Chamberlain et al., 1996; Fang et al., 2005; Geng, 2016): *R. decorum* has a widest distribution range in Hengduan mountain slopes between 2,000 and 3,000 m, and its type specimen was collected from Baoxing, Sichuan province, China. However, *R. vernicosum* has a far smaller distribution than *R. decorum* in the region occurring in the high altitudes between 3,000 and 4,000 m. The type specimen of this species was collected in Kangding city, Sichuan province. Both *R. verruciferum and R. gonggashanense* are confined to one small site, respectively within the range of *R. vernicosum*, and occur in the mountainous slopes similar to *R. vernicosum* (Table 1).

**TABLE 1:** The four species (population) names, location, altitude, site, number of individuals (*N*), and voucher ID of specimen for populations used in the genetic analyses.

**Species/Population**	***N***	**Location (E, N)**	**Altitude(m)**	**Site**	**Voucher ID**
*R. vernicosum*					
E	1	−3.208964, 55.964861	30	Edinburgh	Forrest 5,881
SK	3	99.628242, 27.796356	3,450	Zhongdian	MW 045
PDA	3	99.938295, 27.911097	3,300	Zhongdian	LM 201637
PDB	3	99.938295, 27.911097	3,300	Zhongdian	LM 201638
PDC	3	99.938295, 27.911097	3,300	Zhongdian	LM 201639
XC	3	99.760617, 28.951765	3,975	Xiangcheng	MW 043
XJ	3	102.787806, 30.990656	3,039	Xiaojin	ML 17041
MA	1	102.584489, 31.836386	3,150	Maerkang	ML 170129
MB	3	101.112592, 31.884055	2,949	Maerkang	ML 170128
FRT	3	100.974022, 32.265950	3,288	Rangtang	ML 170133
SGN	3	102.855960, 31.007132	3,262	Xiaojin	ML 17040
YJ	3	100.907159, 29.983389	3,500	Yajiang	MW 037
XDQ	3	101.454461, 30.139214	3,850	Kangding	ML 201815
*R. gonggashanense*					
KA	1	101.759323, 29.531906	3,250	Kangding	MW 025
KB	1	101.544732, 29.471453	3,565	Kangding	MW 034
KC	3	101.759323, 29.531906	3,250	Kangding	MW 026
*R. verruciferum*					
DFA	3	101.219856, 30.889183	3,432	Kangding	MW 019
DFB	3	101.219856, 30.889183	3,432	Kangding	MW 020
DFC	3	101.219220, 30.889717	3,385	Kangding	MW 018
*R. decorum*					
MG	5	103.177912, 28.735226	2,100	Meigu	LJL 201712
BX	1	102.955288, 30.538395	2,228	Baoxing	ML 17058

### Species Sampling and Field Survey

**FIGURE 1:**
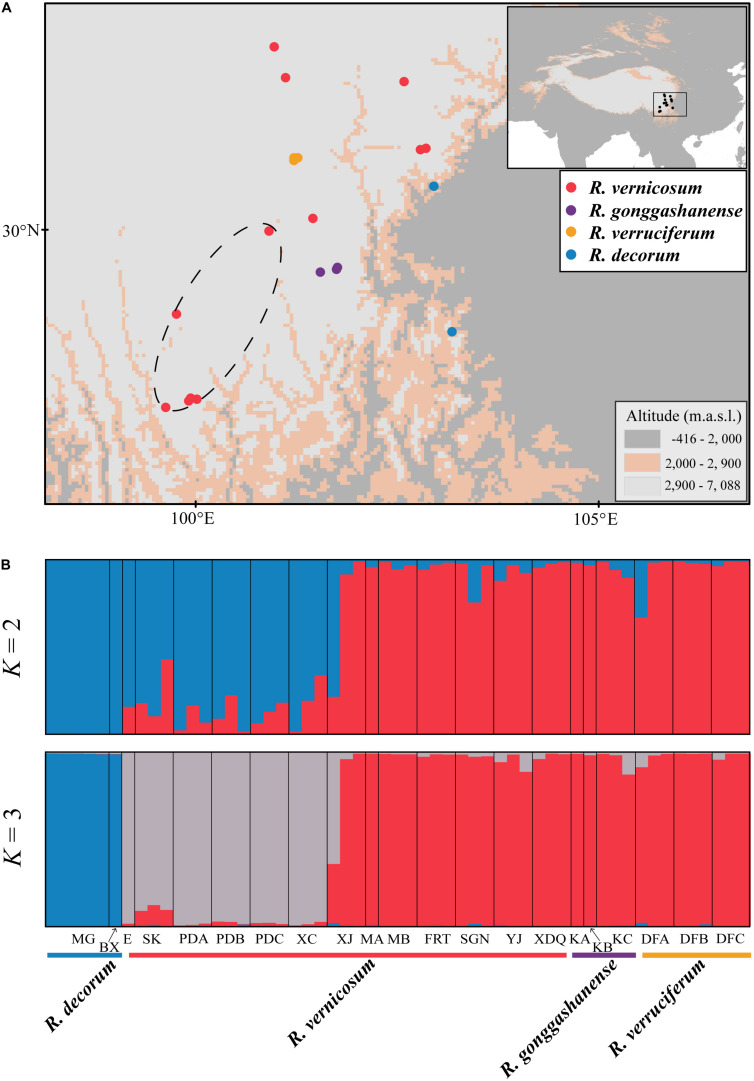
**(A)** The localities of sampling populations for four *Rhododendron* taxa. Four different colored dots represent the populations or individuals corresponding to respective taxa: Red, *R. vernicosum*; Purple, *R. gonggashanense*; Yellow, *R. verruciferum*, and Blue, *R. decorum*. **(B)** The bar plots of ancestry assignments for 55 individuals from 21 populations on STRUCTURE when *K* = 2 and 3, respectively. A likely hybrid group detected in this study is marked by a dark dashed circle in A.

Before field investigations, we examined all specimens of the four species in the species complex kept in the main herbaria in China and the digital images of type specimens from the Jstor database^1^. In addition, type specimens and the collection sites were also checked based on previous publications (Franchet, 1886, 1898; Hu, 1988a,b). Unfortunately, the type specimens of both *R. verruciferum* and *R. gonggashanense* were unavailable in the designated herbaria (SCFI and SZ, respectively) and another possible storage (CDBI). Therefore, we explored and collected the specimens from the type locality of both species: Geka village, Daofu County, and Moxi valley, Gongga Mountain, Kangding city, respectively (Hu, 1988a,b). Though both *R. verruciferum* and *R. gonggashanense* are similar to *R. vernicosum*, nine and five individuals from three populations for each of these two species in type locality and nearby were identified, respectively by morphological characteristics according to the previous descriptions as stated before. As *R. vernicosum* has a relatively widespread distribution in southwest China and occurs at high altitudes > 3,000 m, we collected and selected 35 individuals for this species from 13 populations in the field (Table 1 and Figure 1A) for our analyses. These populations were collected before and specimens from them are characterized by inflorescences with short rachis, pedicles with red gland and sharp leaf apex. In order to avoid mis-identification, we verified the typical morphological traits of flower and fruits of *R. vernicosum* during specimen collection. Only 6 individuals of two populations for *R. decorum* were found based on the previous records of type specimen, and could be readily recognized by its long inflorescence rachis, blunter leaf apex and wide stigmas (Table 1 and Figure 1A). For all available populations of these four species in the field, we collected healthy leaves from mature plants which were at least 50 m apart. Fresh leaves were dried immediately using silica gels for genetic analyses. For each population, we prepared voucher specimens for each mature plant through collecting branchlets with flowers or fruits. These voucher specimens are deposited in the herbarium at Sichuan University, Chengdu, China (SZ). In total, 55 individuals and 46 specimens from 21 typical populations of four species were available for population genetic analysis using SSR markers and morphological data through our field exploration (Table 1).

### Statistical Analyses of Morphological Data

In addition to 46 specimens collected in the field, we further incorporated other 59 previously collected specimens (including type specimens if available) in the herbaria with taxonomic names determined by specialists for final morphological clustering analyses. In total, we used 105 specimens of different individuals (46 ones from our field exploration and 59 ones from the herbaria collected before) from different locations across the distributional ranges of the four species for final morphological data analyses (Supplementary Table 1).

Based on the previous taxonomic treatments and descriptions of these four species (Franchet, 1886, 1898; Hu, 1988a,b), we observed and measured traits of the inflorescent rachis, leaf, pedicle, corolla and stigma for each specimen because they were repeatedly used for taxonomic keys. We extracted 19 morphological traits related to flower and leaf from all 105 specimens. We chose an average of two or three mature leaves to measure all traits. We measured as many flowers as possible from each specimen. Each specific value of each trait was firstly measured and treated using TpsDig v2.05 (Rohlf, 2005). Then the mean values of each individual for the same trait were averaged. These morphological traits (Supplementary Table 1) comprise 13 quantitative traits, including rachis length, pedicle length, corolla length, and width, corolla tube length, corolla base width, corolla length-width ratio, stigma size, petiole length, leaf blade length, leaf blade width, distance from the widest segment of leaf blade to leaf base, leaf length-width ratio, and 6 qualitative traits. Six qualitative traits were processed using the methods of numerical taxonomy (Sokal and Sneath, 1963), including the shape of leaf, the shape of leaf base, the shape of leaf apex, the color of corolla, the color of gland, or glandular hairs in the pedicle and style, the color of spot inner corolla. All parameters of 19 morphological traits were recorded and mean values and standard deviations were calculated for each variable using the software PAST v3.0^2^. Subsequently, we conducted principal coordinates analysis (PCoA) and hierarchical clustering on the standardized variables in the software. We used ANOVA that attached in the software PAST v3.0 to compare variances of the quantitative and qualitative morphological variables between groups detected by PCoA. For computing data in the hierarchical clustering analysis, we chose the Euclidean as similarity index and set the bootstrap values (“Boot *N* = 999”) to establish a clustering dendrogram.

### DNA Extraction and Genetic Clustering Analyses

Genomic DNA were isolated from 20 mg dried leaves using a modified CTAB method for each individual (Doyle and Doyle, 1987). In this study, we used the 15 well-designed polymorphic SSR primers reported previously (Wang et al., 2018). PCR reaction system and process of total DNA were performed by the same way according to our previous study. All PCR amplification products were genotyped by fluorescence capillary electrophoresis using an ABI 3830×l DNA analyzer at Sangon Biological Engineering Technology and Services Co., Ltd., Shanghai, China. Number of alleles per locus (*Na*), the observed heterozygosity (*Ho*) and expected heterozygosity (*He*) were calculated (Supplementary Table 3) using GenALEx v6.501 (Peakall and Smouse, 2012).

We conducted a population genetic clustering analysis of all genetic polymorphisms using the software STRUCTURE v2.3.4 (Pritchard et al., 2000). Twenty replicated runs were performed for each genetic cluster value (*K*) from 1 to 10 with a set of 1200,000 steps in Markov chain following a burn-in of 600,000 steps (Supplementary Table 4). Both the admixture model and correlated allele frequency model were selected in this study. Based on the delta *K*-values (Δ *K*) between successive *K*-values and the log probabilities of data [ln *P* (*D*)], we determined the optimal genetic cluster (K) using a combination of the Pritchard method (Pritchard et al., 2000) and the Evanno method (Evanno et al., 2005) performed in STRUCTURE HARVESTER (Earl and vonHoldt, 2012). For the SSR data, we calculated the genetic differentiation (*F*_*ST*_) between four species using GenALEx v6.501.

## Results

### Morphological Clustering

**FIGURE 2:**
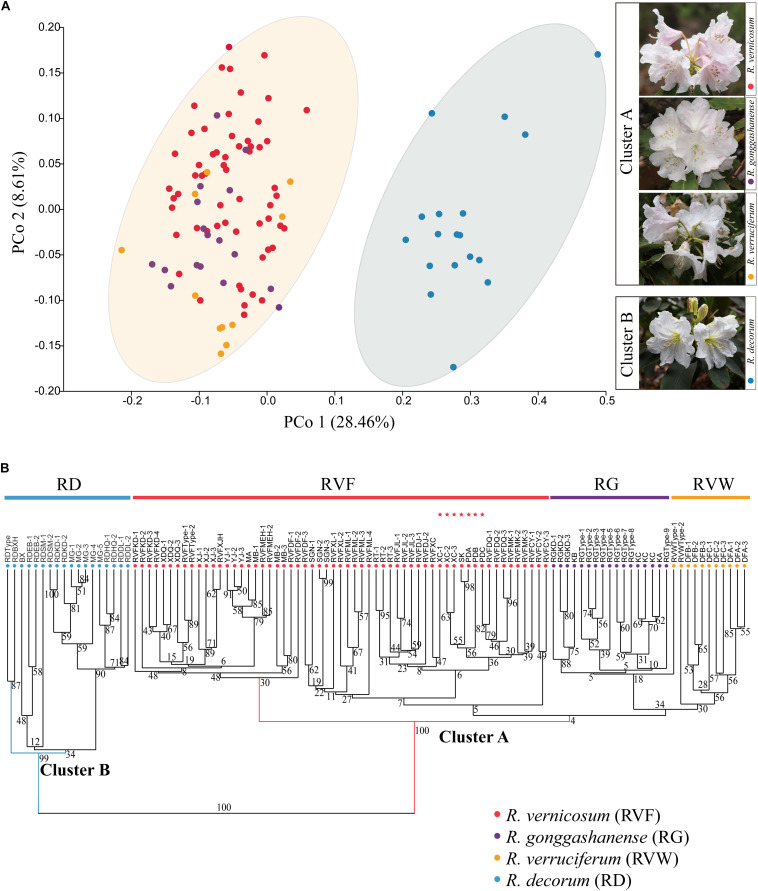
**(A)** Left: Principal co-ordinate analysis (PCoA) of all 105 examined specimens of the four taxa based on the mean value of 19 morphological traits; Right: Inflorescent appearances of four study species, and two clusters identified by the morphological clustering analysis (Cluster A: Red, *R. vernicosum*; Purple, *R. gonggashanense*; Yellow, *R. verruciferum* and Cluster B: Blue, *R. decorum*). **(B)** Hierarchical clustering dendrogram of all 105 examined specimens of four taxa based on the mean value of 19 morphological traits. Numbers above branches are specific values of bootstrap support (BS). These sampled specimens within 6 putative hybrid populations were labeled by red pentagrams. RVF, *R. vernicosum*; RVW, *R. verruciferum*; RG, *R. gonggashanense*; RD, *R. decorum*.

We conducted the PCoA and hierarchical clustering on the observed and measured 19 traits of 105 specimens. The first two principal coordinates (PCo1 and PCo2) in PCoA analysis accounted for 28.46 and 8.61% of the total normalized variance, respectively (Figure 2A). Arrangements of scatterplots based on PCo1 and PCo2 identified two distinct morphological clusters: the first cluster (Cluster A) comprised *R. vernicosum*, *R. gonggashanense*, and *R. verruciferum*, and the other cluster (Cluster B) included *R. decorum*. The test of variances among 19 morphological traits using ANOVA (*P* < 0.05) in PAST showed a significant difference between two groups (Supplementary Table 2). The analysis of hierarchical clustering equally divided these 105 specimens of four species into two main clades, confirming that the clade representing Cluster A could be separated from the other one representing Cluster B. Meanwhile, all individuals of *R. decorum* formed a reasonably well-supported clade [bootstrap support (BS) = 99] and the remaining three species in *R. vernicosum*-*R. decorum* species complex were resolved into a strongly supported (BS = 100) morphological clade (Figure 2B). Moreover, the supports for secondary branches and lower branches under the two main clades were generally weak, especially for a weakly supported branch (BS = 4) comprising *R. gonggashanense* and *R. verruciferum* and a part of individuals within *R. vernicosum* (Figure 2B). Both the analyses by the two approaches recovered similar groupings with all the individuals of *R. vernicosum*, *R. gonggashanense*, and *R. verruciferum* in one cluster (cluster A), and the individuals of *R. decorum* in the other cluster (cluster B). There is no distinct phenotypic difference between *R. vernicosum*, *R. gonggashanense*, and *R. verruciferum*. Morphological traits examined in this study not only included the traits referred in the key to discern different closely related species of the subsection *Fortunea* (Hu and Fang, 1994), but also comprised all phenotypic traits observed in this study, e.g., shapes of corolla and leaf, and rachis length.

### Genetic Differentiation and Clustering Analyses Based on 15 SSR Markers

The clustering analyses using STRUCTURE asscociated with STRUCTURE HARVEST (Earl and vonHoldt, 2012) and a combination of the Pritchard method (Pritchard et al., 2000) and the Evanno method (Evanno et al., 2005) revealed that the most likely number of genetic clusters was 3 (*K* = 3) (Supplementary Figures 1,2), dividing the 55 sampled individuals from 21 populations into three genetic groups (Figure 1B, *K* = 3). The first cluster comprised all the individuals of *R. decorum*, the second cluster comprised six populations of *R. vernicosum* and the third cluster comprised seven populations of *R. vernicosum* and all individuals of *R. gonggashanense* and *R. verruciferum*. Interestingly, the second cluster also included the genetic introgression from the third cluster. When the value of *K* was 2 (*K* = 2), two relatively pure genetic groups were identified for *R. decorum* and *R. vernicosum-R. gonggashanense*-*R. verruciferum* while the second group comprised six populations of *R. vernicosum* with genetic mixtures from the other two genetic groups particularly from the *R. decorum* group (Figure 1B).

Genetic differentiation between two genetic groups representing six and seven populations, respectively, from *R. vernicosum* (*F_*ST*_* = 0.075) was detected (Supplementary Table 5). However, genetic differentiations between seven populations of *R. vernicosum* including the type locality (XDQ) and *R. gonggashanense* and *R. verruciferum* were obviously low (*F_*ST*_* = 0.054 and 0.052, respectively). In addition, the genetic differentiation between *R. gonggashanense* and *R. verruciferum* was similarly low (*F_*ST*_* = 0.05).

## Discussion

Our findings based on the statistical analyses of morphological traits from four species, suggest that both *R. verruciferum* and *R. gonggashanense* should be incorporated into *R. vernicosum*. In addition, morphological traits that were used to differentiate *R. gonggashanense R. vernicosum*, and *R. verruciferum*, including gland or glandular hairs color in pedicel, style and ovary, and the shape or size of the leaf and corolla (Hu, 1988a,b), seemed to be highly variable between individuals from different populations within the widespread *R. vernicosum* based on the analyses of morphological clustering. However, the morphological traits could be employed to distinguish *R. decorum* from the other three species. These traits include the ones used in traditional key, and newly examined ones as well, such as rachis length, petiole length, length and width of leaf blade, distance from the widest segment of leaf blade to leaf base, leaf length and width ratio, shape of leaf base, stigma size, and the color of blotch or spots inner corolla. This was further supported by the population genetic data using 15 SSR markers. Both the two species and seven populations of *R. vernicosum* comprised a well-delimitated genetic group, distinctly different from *R. decorum*. In addition, the third genetic group was identified to comprise the remaining six populations of *R. vernicosum* and show the genetic mixture from the other two groups. The genetic ancestry of this group from *R. decorum* is more than that from the other genetic group of *R. vernicosum* although both groups of *R. vernicosum* were placed in the same morphological cluster (Figure 2), which seemed to be a likely hybrid group with more genetic ancestry from *R. decorum*.

### Taxonomic Reduction of *R. verruciferum* and *R. gonggashanense* to *R. vernicosum*

*Rhododendron vernicosum* and *R. decorum* were published by Franchet (1886, 1898) in the late nineteenth century and their type specimens were collected from Kangding, Sichuan, China and Baoxing county, Sichuan, respectively. However, *R. verruciferum* and *R. gonggashanense* were described based on specimens from two small sites (Daofu, Sichuan province, and Kangding, Sichuan province, respectively.) (Hu, 1988a,b). Both species were compared with *R. decorum* and the major morphological differences between them were ascribed to leaf shape, flower size and the gland or gland hairs in the pedicle or style. Examination of type specimens with protologs, well-determinant specimens in the herbaria and the specimens collected in the field investigations covering the distributional ranges of the four taxa revealed that *R. verruciferum* and *R. gonggashanense* resembled *R. vernicosum* other than *R. decorum*. All morphological variations of specimens of *R. verruciferum* and *R. gonggashanense* collected from the type locality and nearby areas were covered by those of *R. vernicosum* (Figure 2). The sampled specimens from these three species comprised one morphological entity (Cluster A), distinct from the cluster comprising the specimens of *R. decorum* (Cluster B). In fact, these two groups (Cluster A and B) were distinguished by numerous characters, including the color of spot in inner corolla and corolla color, the length of rachis and pedicle, and the distributional altitudes. In addition, both *R. verruciferum* and *R. gonggashanense* were recorded to confined in two very small sites in southwest China. The distributional range of *R. vernicosum* obviously encompasses these two small sites, as *R. vernicosum* is relatively widespread in southwest China (Figure 1A).

Our population genetic analyses of the four species based on 15 SSR markers revealed that *R. verruciferum* and *R. gonggashanense* were likely described based on the intraspecific variations of the widespread *R. vernicosum.* Two distinct genetic groups were recovered for *R. vernicosum* and *R. decorum*, respectively, when the population genetic group (*K*) is 2 (Figure 1B, *K* = 2). All individuals of *R. verruciferum* and *R. gonggashanense* were placed in the genetic group represented by seven populations of *R. vernicosum*. In the *R. vernicosum* group, the individuals collected from the type locality (Kangding, Sichuan) of *R. vernicosum* were included. When the optimal genetic cluster is 3 (Figure 1B, *K* = 3), another genetic group was identified for the six populations of *R. vernicosum*, while still retaining *R. verruciferum* and *R. gonggashanense* within the *R. vernicosum* genetic group. All of these findings suggest that *R. verruciferum* and *R. gonggashanense* are conspecific to *R. vernicosum*. The taxonomically revised *R. vernicosum* should comprise at least seven populations of *R. vernicosum, R. verruciferum*, and *R. gonggashanense*.

### Putative Hybrid of *R. vernicosum* and *R. decorum*

We identified three genetic groups by genotyping 21 populations of the four species. Especially, we recovered one hybrid genetic group between the other two groups represented by *R. decorum* and *R. vernicosum-R. gonggashanense*-*R. verruciferum* populations. When *K* = 2 (Figure 1B), the genetic mixture was obvious for this group but with more genetic ancestry from *R. decorum*. This group comprises six populations of *R. vernicosum*, whose morphological traits examined herein showed high similarity with the *R. vernicosum-R. gonggashanense*-*R. verruciferum* genetic group without distinct phenotypic differences (Figure 2). The genetic ancestry from *R. vernicosum*, although less than that from *R. decorum*, might have contributed greatly to this similarity in the examined morphological traits. In addition, when *K* = 3, this hybrid genetic group comprised one distinct group from the other two groups (Figure 1B), suggesting that this hybrid group may have developed into an independently evolving lineage. Based on the distributions of the three genetic groups, we also found that they occupied different distributional ranges (Figure 1A).

Because of high outcrossing abilities and incomplete reproductive isolations, interspecific hybridizations occur frequently in the genus *Rhododendron* (Ma et al., 2010; Milne et al., 2010; Marczewski et al., 2015; Yan et al., 2015; Wang et al., 2018). These interspecific hybridizations produce hybrids with a combination of morphological traits of two parental species or cryptic genetic introgressions occur in one or two parental species without morphological changes. However, all these hybrids or introgressed individuals occur in the overlapping regions of the two parental species without distinct geographical distributions or niches. The present hybrid genetic group found between the two pure *R. decorum* and *R. vernicosum* groups occupies a different distributional range than the ones occupied by the other two species. It may likely represent a new hybrid species as other hybrid species reported previously by some studies in southwest China (Sun et al., 2013; Liu et al., 2014; Jia et al., 2015). However, this hybrid genetic group does not co-occur with pure *R. decorum* and *R. vernicosum* group. It may might result from long-distance seed dispersals after hybridization between two parents because the winged *Rhododendron* seeds can be dispersed by wind (Berg, 1983; Nathan et al., 2008). Alternatively, this hybrid group had co-existed with two parents during the initial hybridization process, but gradually replaced them because of the hybrid heterosis as suggested before (Moore, 1977; Arnold, 1997; Milne et al., 2003). However, further studies should include more morphological traits than those examined here in order to accurately verify the true identity of the hybrid group. It is expected that this hybrid group may have a combination of the differentiated morphological traits between other two, which were neglected or not sampled here and in previous taxonomic studies (Franchet, 1886, 1898; Hu, 1988a,b; Hu and Fang, 1994; Fang et al., 2005; Geng, 2016), for example, fruits or style traits.

## Conclusion

In conclusion, our findings on the *R. vernicosum*-*R. decorum* species complex based on statistical morphological analyses and population genetic data from SSR markers suggest that *R. verruciferum* and *R. gonggashanense* should be taxonomically reduced to *R. vernicosum*. In addition, some populations placed under *R. vernicosum* based on the currently used morphological traits for species delimitations may represent one likely hybrid species because this group comprises multiple populations with a distinct distribution. Such a hybrid group may have started independent evolution as a separate lineage. The genetic ancestry of this hybrid species is derived more from *R. decorum* than from *R. vernicosum* although it is morphologically similar to *R. vernicosum*. Further studies and confirmations are needed based on the detailed examination of more morphological traits at the population level and genomic analyses of the currently sampled populations as well as more from the total distributional ranges of the three identified genetic groups. Only after rigorous analyses, can the taxonomic revisions be correctly performed for the *R. vernicosum*-*R. decorum* species complex.

## Data Availability Statement

The raw data supporting the conclusions of this article will be made available by the authors, without undue reservation, to any qualified researcher.

## Author Contributions

JL supervised the writing. XM collected materials, designed experiment, and wrote the manuscript. JW extracted DNA and analysed SSR data. NS revised the manuscript. YM provided the ideas. All authors contributed to the article and approved the submitted version.

## Conflict of Interest

The authors declare that the research was conducted in the absence of any commercial or financial relationships that could be construed as a potential conflict of interest.

## Footnotes

1 http://plants.jstor.org/

2 http://folk.uio.no/ohammer/past/

## Supplementary Material

Supplementary Figure 1 Estimation of the log probability of data [ln *P* (*D*)] for each *K*.

Supplementary Figure 2 Estimation of the likely genetic cluster of 55 individuals from 21 populations of four taxa for *K* ranging from one to nine by Δ *K*-values.

Supplementary Table 1 The statistical data of 19 morphological traits for 105 specimens from four taxa used in software PAST.

Supplementary Table 2 The test of variances among 19 morphological traits between groups using ANOVA in software PAST.

Supplementary Table 3 Genetic properties of 15 well-designed markers for four *Rhododendron* species.

Supplementary Table 4 The summary of simulations of the STURCTURE in our study.

Supplementary Table 5 Genetic differentiation between four *Rhododendron* species.
