# Supplementary figures and images for: Species Identification in the Rhododendron vernicosum–R. decorum Species Complex (Ericaceae)
Source: Front Plant Sci. 2021 Jan 28;12:608964. doi: 10.3389/fpls.2021.608964 (PMC7876077; doi:10.3389/fpls.2021.608964)

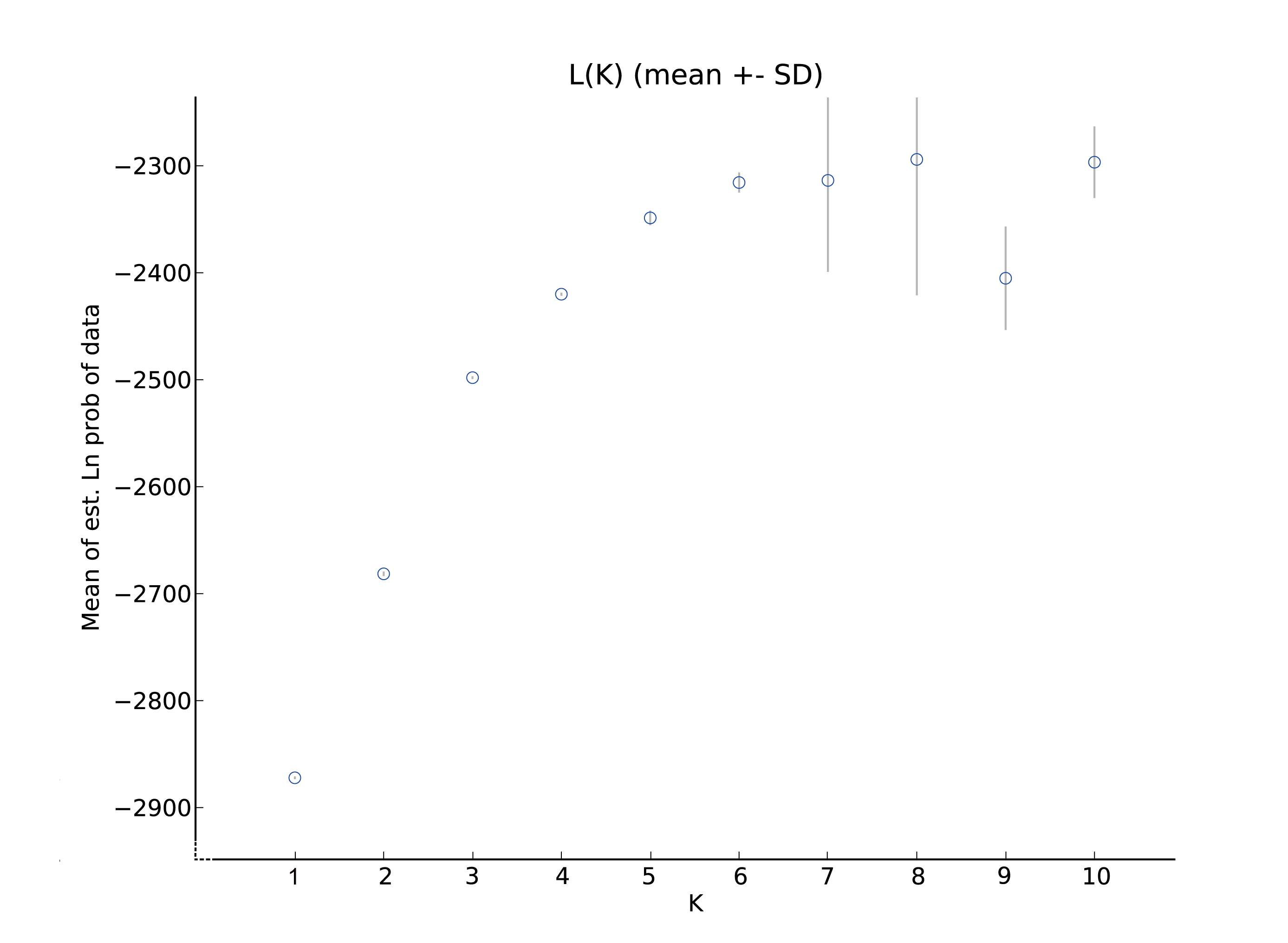

Supplement: Supplementary Figure 1 — Estimation of the log probability of data [ln P (D)] for each K. [file Image_1.JPEG]

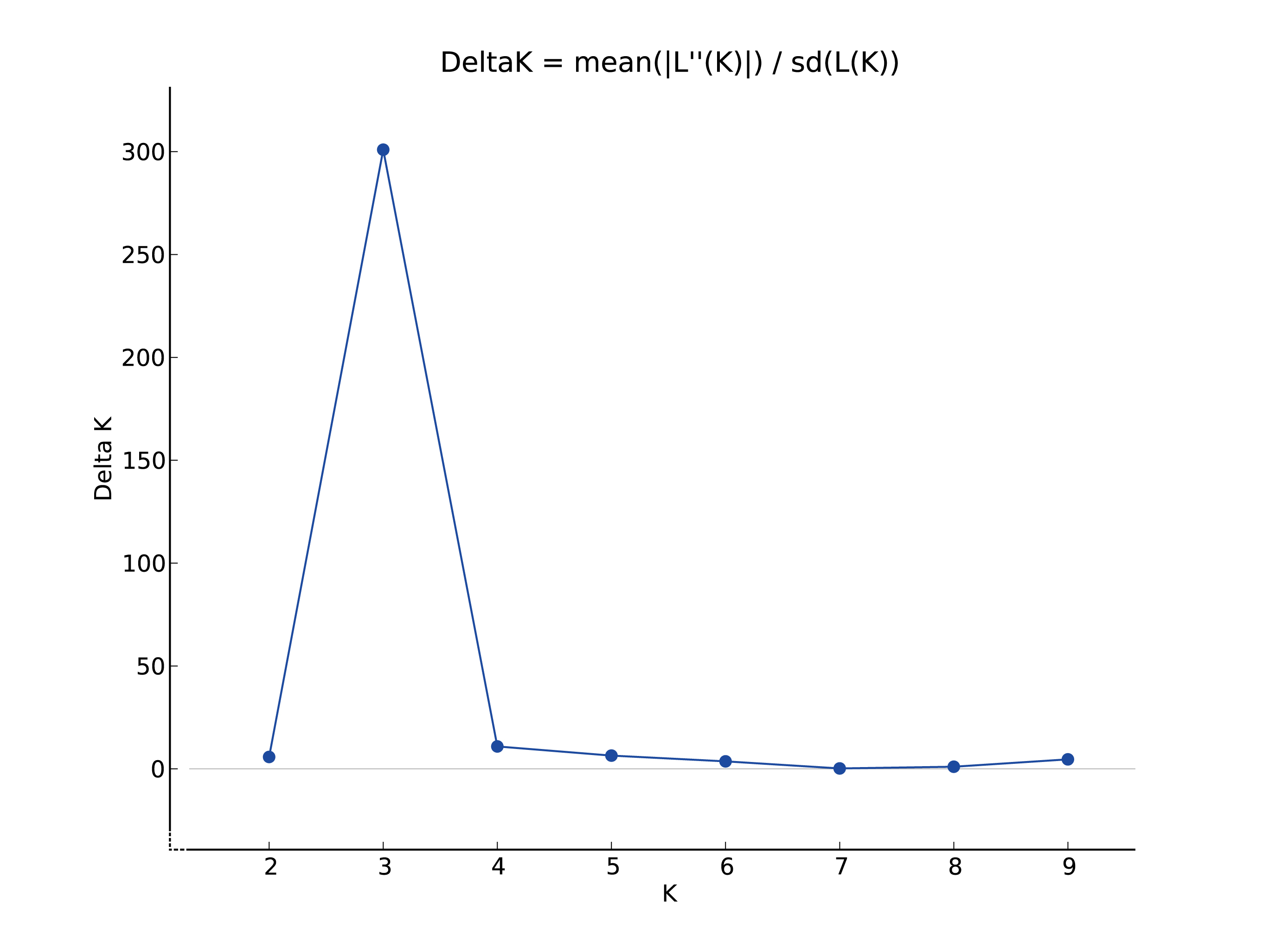

Supplement: Supplementary Figure 2 — Estimation of the likely genetic cluster of 55 individuals from 21 populations of four taxa for K ranging from one to nine by Δ K-values. [file Image_2.JPEG]
