# Supplementary material for: Species Identification in the Rhododendron vernicosum–R. decorum Species Complex (Ericaceae)
Source: Front Plant Sci. 2021 Jan 28;12:608964. doi: 10.3389/fpls.2021.608964 (PMC7876077; doi:10.3389/fpls.2021.608964)
Supplement: Supplementary Table 1 — The statistical data of 19 morphological traits for 105 specimens from four taxa used in software PAST. [file Table_1.docx]

| **Species/Population** | ***N*** | **Location (E; N)** | | **Altitude(m)** | **Site** | **Voucher ID** |
| --- | --- | --- | --- | --- | --- | --- |
| *R. vernicosum* |  |  |  | |  |  |
| E | 1 | -3.208964; 55.964861 | 30 | | Edinburgh | Forrest 5881 |
| SK | 3 | 99.628242; 27.796356 | 3,450 | | Zhongdian | MW 045 |
| PDA | 3 | 99.938295; 27.911097 | 3,300 | | Zhongdian | LM 201637 |
| PDB | 3 | 99.938295; 27.911097 | 3,300 | | Zhongdian | LM 201638 |
| PDC | 3 | 99.938295; 27.911097 | 3,300 | | Zhongdian | LM 201639 |
| XC | 3 | 99.760617; 28.951765 | 3,975 | | Xiangcheng | MW 043 |
| XJ | 3 | 102.787806; 30.990656 | 3,039 | | Xiaojin | ML 17041 |
| MA | 1 | 102.584489; 31.836386 | 3,150 | | Maerkang | ML 170129 |
| MB | 3 | 101.112592; 31.884055 | 2,949 | | Maerkang | ML 170128 |
| FRT | 3 | 100.974022; 32.265950 | 3,288 | | Rangtang | ML 170133 |
| SGN | 3 | 102.855960; 31.007132 | 3,262 | | Xiaojin | ML 17040 |
| YJ | 3 | 100.907159; 29.983389 | 3,500 | | Yajiang | MW 037 |
| XDQ | 3 | 101.454461; 30.139214 | 3,850 | | Kangding | ML 201815 |
| *R. gonggashanense* |  |  |  | |  |  |
| KA | 1 | 101.759323; 29.531906 | 3,250 | | Kangding | MW 025 |
| KB | 1 | 101.544732; 29.471453 | 3,565 | | Kangding | MW 034 |
| KC | 3 | 101.759323; 29.531906 | 3,250 | | Kangding | MW 026 |
| *R. verruciferum* |  |  |  | |  |  |
| DFA | 3 | 101.219856; 30.889183 | 3,432 | | Kangding | MW 019 |
| DFB | 3 | 101.219856; 30.889183 | 3,432 | | Kangding | MW 020 |
| DFC | 3 | 101.219220; 30.889717 | 3,385 | | Kangding | MW 018 |
| *R. decorum* |  |  |  | |  |  |
| MG | 5 | 103.177912; 28.735226 | 2,100 | | Meigu | LJL 201712 |
| BX | 1 | 102.955288; 30.538395 | 2,228 | | Baoxing | ML 17058 |

**Tables**

Table 1: The four species (population) names, location, altitude, site, number of individuals (*N*) and voucher ID of specimen for populations used in the genetic analyses.
