# Supplementary material for: Species Identification in the Rhododendron vernicosum–R. decorum Species Complex (Ericaceae)
Source: Front Plant Sci. 2021 Jan 28;12:608964. doi: 10.3389/fpls.2021.608964 (PMC7876077; doi:10.3389/fpls.2021.608964)
Supplement: Supplementary Table 4 — The summary of simulations of the STURCTURE in our study. [file Table_4.DOCX]

Supplementary Table S3 Genetic properties of 15 well-designed markers of four *Rhododendron* species.

| **Locus ID** | **Sample size** | **Na** | **He** | **Ho** | **HWE *p*** |
| --- | --- | --- | --- | --- | --- |
| RhSSR_03 | 55 | 14 | 0.636364 | 0.73157 | <0.001 |
| RhSSR_04 | 55 | 10 | 0.581818 | 0.675372 | <0.001 |
| RhSSR_05 | 44 | 23 | 0.477273 | 0.893337 | <0.001 |
| RhSSR_06 | 55 | 13 | 0.581818 | 0.721322 | <0.001 |
| RhSSR_07 | 55 | 5 | 0.127273 | 0.390248 | <0.001 |
| RhSSR_08 | 55 | 8 | 0.418182 | 0.550744 | <0.001 |
| RhSSR_09 | 54 | 19 | 0.574074 | 0.90415 | <0.001 |
| RhSSR_10 | 55 | 7 | 0.181818 | 0.278843 | <0.001 |
| RhSSR_11 | 52 | 10 | 0.211538 | 0.838572 | <0.001 |
| RhSSR_12 | 55 | 5 | 0.127273 | 0.459669 | <0.001 |
| RhSSR_13 | 55 | 20 | 0.6 | 0.766116 | <0.001 |
| RhSSR_14 | 55 | 8 | 0.454545 | 0.58562 | <0.001 |
| RhSSR_15 | 53 | 28 | 0.867925 | 0.94037 | ns |
| RhSSR_16 | 54 | 6 | 0.314815 | 0.694959 | <0.001 |
| RhSSR_19 | 55 | 15 | 0.472727 | 0.821322 | <0.001 |
