# Supplementary material for: Species Identification in the Rhododendron vernicosum–R. decorum Species Complex (Ericaceae)
Source: Front Plant Sci. 2021 Jan 28;12:608964. doi: 10.3389/fpls.2021.608964 (PMC7876077; doi:10.3389/fpls.2021.608964)
Supplement: Supplementary file 8 [file Table_6.DOCX]

|  | *R. decorum* | *R. vernicosum*  (Six populations) | *R. vernicosum*  (Seven populations) | *R. gonggashanense* | *R. verruciferum* |
| --- | --- | --- | --- | --- | --- |
| *R. decorum* | 0.000 | —— | —— | —— | —— |
| *R. vernicosum*  (Six populations) | 0.173 | 0.000 | —— | —— | —— |
| *R. vernicosum*  (Seven populations) | 0.208 | 0.075 | 0.000 | —— | —— |
| *R. gonggashanense* | 0.228 | 0.092 | 0.054 | 0.000 | —— |
| *R. verruciferum* | 0.248 | 0.104 | 0.052 | 0.050 | 0.000 |

Supplementary Table S5 Genetic differentiation between four *Rhododendron* species
