# Supplementary material for: Species Identification in the Rhododendron vernicosum–R. decorum Species Complex (Ericaceae)
Source: Front Plant Sci. 2021 Jan 28;12:608964. doi: 10.3389/fpls.2021.608964 (PMC7876077; doi:10.3389/fpls.2021.608964)
Supplement: Supplementary file 9 [file Data_Sheet_1.PDF]

CLUMPAK main pipeline - Job 1609158491 summary

Major modes for the uploaded data:

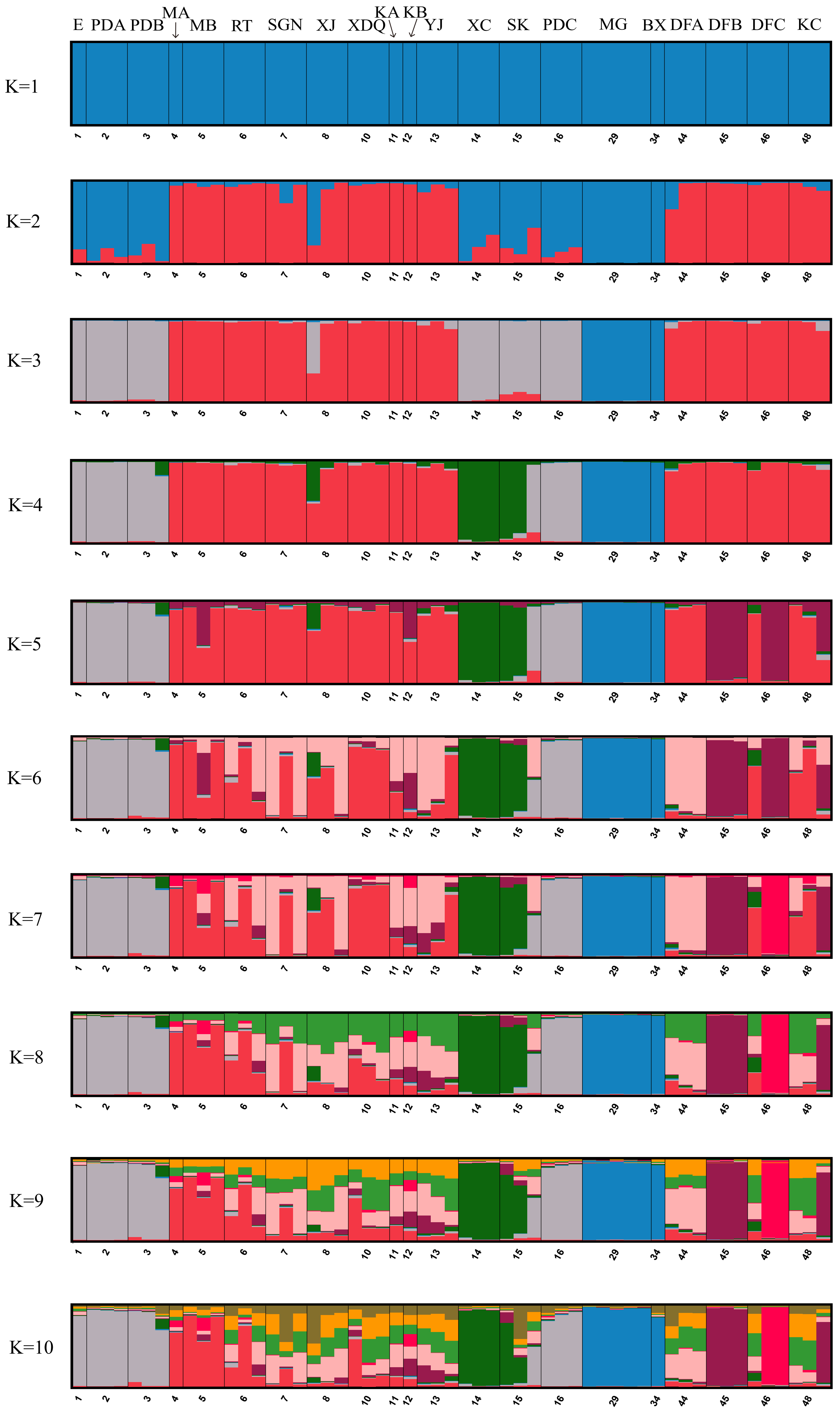

Minor modes for the uploaded data:

K=5 MinorCluster1

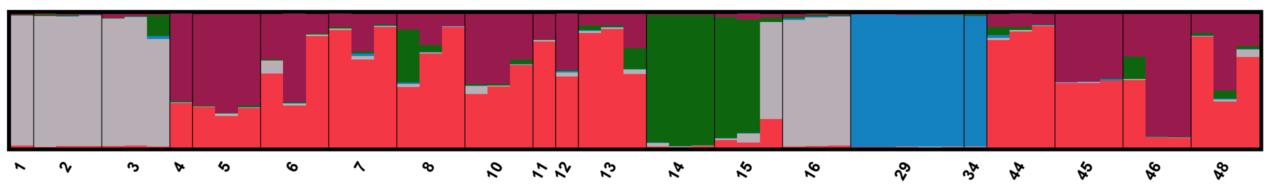

K=6 MinorCluster1

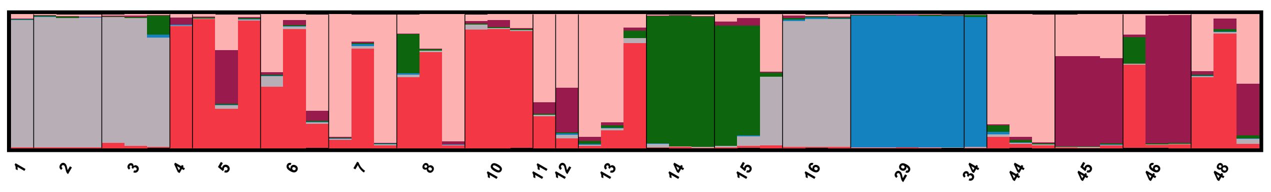

K=7 MinorCluster1

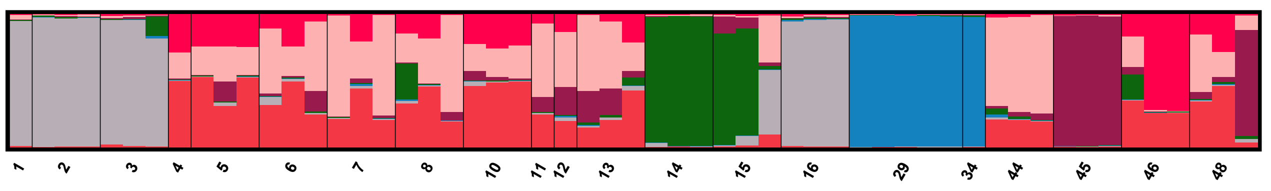

K=7 MinorCluster2

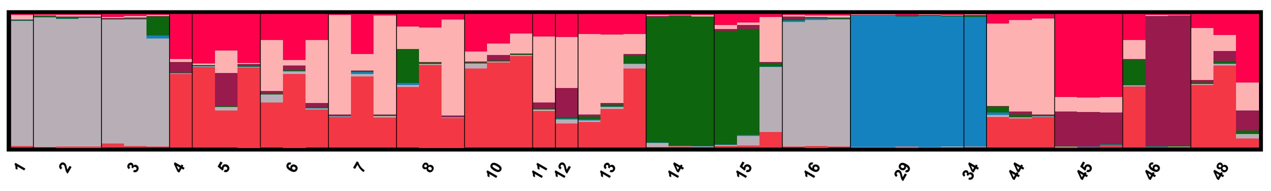

K=8 MinorCluster1

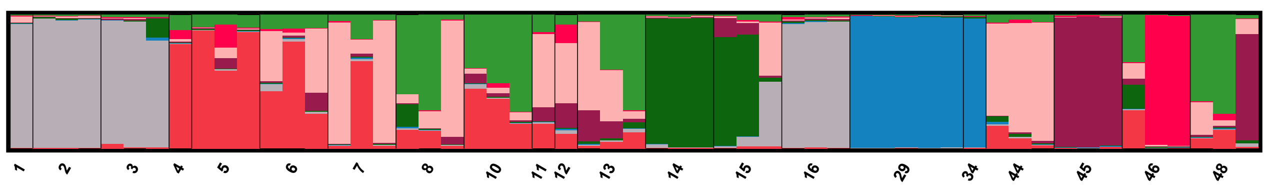

K=8 MinorCluster2

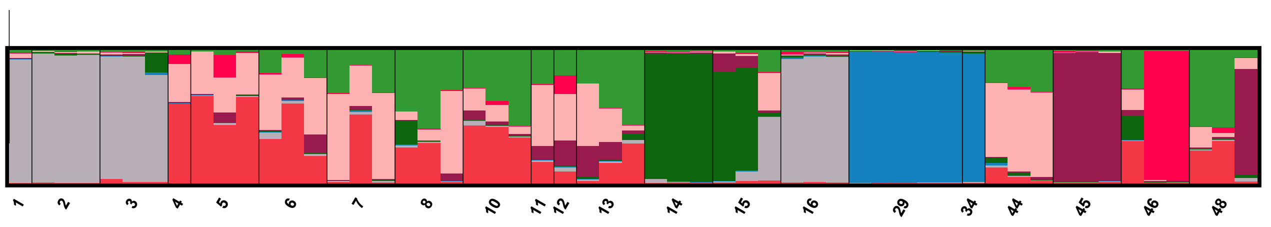

Division of runs by mode:

K=1 20/20
K=2 20/20
K=3 20/20
K=4 20/20
K=5 13/20, 7/20
K=6 11/20, 7/20
K=7 15/20, 2/20, 2/20
K=8 10/20, 7/20, 3/20
K=9 20/20
K=10 19/20
